# Endoscopic management of proximally migrated pancreatic duct stents: A case series of three patients and clinical insights

**DOI:** 10.1055/a-2888-9289

**Published:** 2026-06-29

**Authors:** Jieyao Cheng, Xiue Yan, Hong Chang, Yingchun Wang, Yaopeng Zhang, Wenzheng Liu, Yonghui Huang

**Affiliations:** 1 Department of Gastroenterology 589702 Beijing Tsinghua Changgung HospitalBeijingBeijingChina; 2 Department of Gastroenterology 66482 Peking University Third HospitalBeijingBeijingChina

Pancreatic duct stenting is essential for preventing post-endoscopic retrograde cholangiopancreatography (ERCP) pancreatitis and treating ductal strictures. 1​ Proximal stent migration occurs in 5–6% of cases and may lead to severe complications if not promptly retrieved. 2​ We present three complex cases successfully managed with endoscopic techniques.

**Case 1** : A 56-year-old man presented with back pain 2 months after ERCP at another hospital, where a straight pancreatic stent (7Fr) had migrated proximally. Pancreatography revealed the stent embedded in a stricture within the pancreatic head. After bougie dilation (7Fr and 8.5Fr), conventional devices failed. The stent was successfully extracted using SpyBite biopsy forceps ( Video 1 ).

**Video 1** Endoscopic management of proximally migrated pancreatic stents in three challenging cases: SpyBite for the stricture, SpyGlass‑guided side‑hole grasping for the branch duct, and minor papilla approach with a perpendicular angle for grasping.


**Case 2** : A 59-year-old man developed pancreatitis due to proximal stent migration 4 weeks after ERCP. Retrieval attempts elsewhere had failed. SpyGlass pancreatoscopy showed the stent lodged in a branch duct. Forceps attempts were unsuccessful due to slipping, and precise grasping of the stent's side hole under direct visualization enabled successful retrieval ( Video 1 ).

**Case 3** : A 33-year-old man with incomplete pancreas divisum underwent pancreatic stenting (5Fr). Within 24 hours, the stent migrated into the pancreatic body. Retrieval via the major papilla failed. After cannulating and dilating the minor papilla, foreign body forceps achieved a perpendicular angle for grasping ( **Figs** . **​​​​​​​** 1and 2 ).

**Fig. 1:**
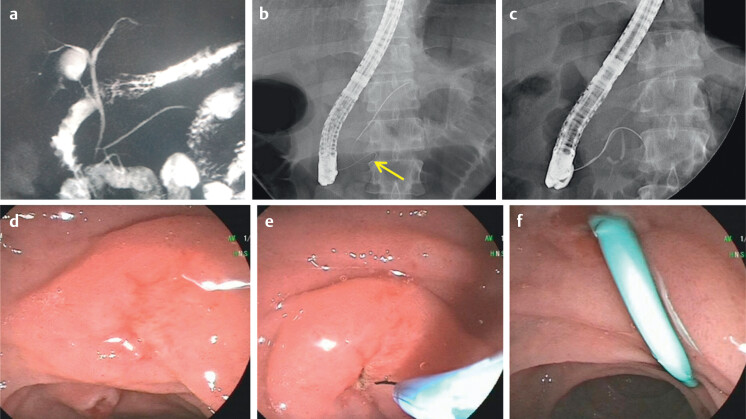
Endoscopic management of a patient with incomplete pancreas divisum. ( **a** ) MRCP revealed a reverse α‑type pancreatic duct with incomplete pancreas divisum. ( **b** ) X‑ray confirms the same anatomy (arrow). ( **c** ) Pancreatography shows a tortuous, looped ventral duct with mild dilation, angled confluence with the dorsal duct, and irregular narrowing of the accessory duct. ( **d** ) The minor papilla orifice is stenotic. ( **e** ) A needle‑knife is used for minor papilla sphincter pre-cut to establish drainage. ( **f** ) A pancreatic stent is placed via the major papilla to prevent post‑ERCP pancreatitis.

**Fig. 2:**
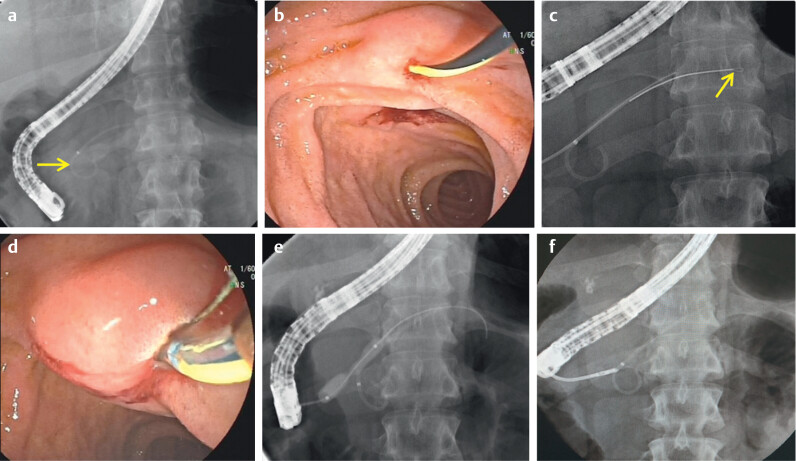
Endoscopic retrieval of a migrated pancreatic stent via the minor papilla in a patient with incomplete pancreas divisum. ( **a** ) The migrated stent (arrow) in the pancreatic body. ( **b** ) Minor papilla cannulation. ( **c** ) Guidewire looping (arrow) fails to capture the stent. ( **d** ) The minor papilla is enlarged with a papillotome. ( **e** ) Balloon dilation of the minor papilla and the stricture at the ventral–dorsal duct junction. ( **f** ) Forceps grasping the stent at a perpendicular angle.

Proximal stent migration poses challenges depending on the location. According to Matsumoto's classification, 3​ Type D (branch duct embedding) is most difficult. For Type C migration (above a stricture), stricture dilation followed by SpyBite forceps retrieval is recommended, as its serrated jaw provides superior gripping strength. For Type D, SpyGlass-guided side hole grasping is preferred. 4​ ​5​ When major papilla access fails due to tortuous anatomy, the minor papilla route with a perpendicular forceps angle should be attempted.

Individualized strategies based on anatomical subtype are essential. For Type D migration, pancreatoscopy-guided side hole grasping is the preferred technique.

Endoscopy_UCTN_Code_TTT_1AR_2AZ

## References

[1] Tarnasky P R Palesch Y Y Cunningham J T Pancreatic stenting prevents pancreatitis after biliary sphincterotomy in patients with sphincter of Oddi dysfunction Gastroenterol 1998 115 1518 1524 10.1016/s0016-5085(98)70031-9 9834280

[2] Johanson J F Schmalz M J Geenen J E Incidence and risk factors for biliary and pancreatic stent migration Gastrointest Endosc 1992 38 341 346 1607087 10.1016/s0016-5107(92)70429-5

[3] Matsumoto K Katanuma A Maguchi H Endoscopic removal technique of migrated pancreatic plastic stents J Hepatobiliary Pancreat Sci 2014 21 E34 E40 24535753 10.1002/jhbp.94

[4] Maydeo A Kwek A Bhandari S SpyGlass pancreatoscopy-guided cannulation and retrieval of a deeply migrated pancreatic duct stent Endoscopy 2011 43 E137 E138 21425014 10.1055/s-0030-1256205

[5] Bhandari S Sharma A Bathini R Endoscopic management of internally migrated pancreatic duct stents (with video) Indian J Gastroenterol 2016 35 91 100 27030246 10.1007/s12664-016-0638-z

